# Persistence on biologic DMARD monotherapy after achieving rheumatoid arthritis disease control on combination therapy: retrospective analysis of corrona registry data

**DOI:** 10.1007/s00296-020-04667-5

**Published:** 2020-09-02

**Authors:** Dimitrios A. Pappas, Heather J. Litman, Tamara Lesperance, Greg Kricorian, Elaine Karis, Sabrina Rebello, Winnie Hua, Neil A. Accortt, Scott Stryker

**Affiliations:** 1 Department of Epidemiology and Outcomes Research, Corrona, LLC, Waltham, MA USA; 2 Columbia University College of Physicians and Surgeons, Columbia University, 630 W 168th St, P&S Building, Suite 10-445, New York, NY 10032 USA; 3 Department of Biostatistics, Corrona, LLC, Waltham, MA USA; 4 DOCS Global, Inc., North Wales, PA USA; 5 US Medical Affairs, Amgen Inc., Thousand Oaks, CA USA; 6 Center for Observational Research, Amgen Inc., Thousand Oaks, CA USA

**Keywords:** TNF inhibitors, Etanercept, Methotrexate, Antirheumatic drugs, Disease-modifying antirheumatic drugs, Arthritis, Rheumatoid

## Abstract

Biological disease-modifying antirheumatic drugs (bDMARDs) monotherapy may enhance adherence and decrease adverse events compared to combination therapy with conventional synthetic DMARDs (csDMARDs); however, persistence with bDMARD monotherapy has not been extensively studied. We explore persistence of etanercept monotherapy and monotherapy with other tumor necrosis factor inhibitors (TNFis) among patients first achieving remission/low disease activity (LDA) while on combination therapy with csDMARDs and a TNFi. Using Corrona registry data, the percentage of patients persistent with the index TNFi (etanercept versus other TNFis) over 6 and 12 months was determined. Factors influencing persistence and treatment patterns at 6 and 12 months were examined. Among 617 eligible patients, 56% of 182 patients on etanercept and 45% of 435 patients on other TNFis persisted with monotherapy at 6 months, 46% and 33%, respectively, at 12 months. Across first-line and subsequent biologic DMARDs, etanercept persistence was greater than other TNFi persistence by 10.8% (95% CI 2.1%, 19.6%) at 6 months and 11.4% (95% CI 0.9%, 21.9%) at 12 months. Patients on other TNFis were more likely to require reintroduction of csDMARD after 6 months (45% versus 35% for etanercept). Remission was the key predictor of persistence for both etanercept and other TNFi monotherapies. This retrospective, cohort study of registry data reflecting real-world practice indicates patients who achieve remission/LDA with combination csDMARD and TNFi therapy may successfully transition to TNFi monotherapy. Patients on etanercept monotherapy experienced greater persistence and less frequent reintroduction of a csDMARD than was observed for patients on other TNFi monotherapies.

## Introduction

Rheumatoid arthritis (RA) is a chronic, systemic autoimmune disease affecting the synovial lining of the joints [1], with an estimated prevalence of 0.5–1.0% [2]. In the United States, RA has an estimated annual incidence of 41 cases per 100,000 persons, is estimated to affect approximately 1.5 million adults [3], and is associated with extensive morbidity and reduced quality of life [4]. When the disease is insufficiently treated, joint damage and extra-articular manifestations can result, accompanied by irreversible disability [1, 2].

There is currently no known cure for RA [1]; nonetheless, the current European League Against Rheumatism (EULAR), American College of Rheumatology (ACR), and Asia Pacific League of Associations for Rheumatology (APLAR) treatment guidelines all recognize the achievement of remission or low disease activity (LDA) as a realistic goal for treat-to-target strategies [5–7]. Guidelines recommend conventional synthetic disease-modifying agents (csDMARDs) as first-line treatment for RA [5–7]. For patients not achieving sufficient disease control with csDMARDs alone, the addition of biologic DMARDs (bDMARDs) is recommended [5–7]. Although the guidelines are clear about the role of combination therapy, there is less clarity about the treatment approach once remission or LDA has been achieved [5–7]. For patients in sustained remission, no specific guidance is provided for tapering or discontinuing treatment in patients on combination therapy. Given that the use of multiple therapies may compromise patient adherence [8], and that 10–37% of patients with RA cannot tolerate methotrexate [9, 10], the option of monotherapy with bDMARDs is of interest, particularly as adherence to bDMARDs appears to be higher than adherence to csDMARDs, such as methotrexate [11]. Monotherapy with bDMARDs may enhance adherence and decrease the chances of adverse events compared to combination therapy with csDMARDs, such as methotrexate [12]. However, the persistency of TNFi monotherapy after achieving disease control on combination therapy has not been extensively investigated.

TNFis are the most commonly used bDMARDs for the treatment of RA. TNFis include monoclonal antibodies against TNF and etanercept [5–7]. Etanercept is a dimeric fusion protein consisting of the binding portion of the TNF receptor linked to human immunoglobulin [13] and does not lyse cells expressing TNF-α [14]. Unlike monoclonal antibodies to TNF [15, 16], etanercept has not been associated with the formation of neutralizing antidrug antibodies [17]. Methotrexate has been found to reduce the development of antidrug antibodies to TNFis [18]. Since etanercept does not induce neutralizing antibodies like monoclonal TNFis, the use of methotrexate with etanercept is not necessary to prevent the formation of neutralizing antidrug antibodies, and for this reason, the continued use of methotrexate in patients treated with etanercept may not be needed in patients who have achieved good disease control.

The primary objective of this retrospective, cohort study and analysis of existing data from the Corrona registry reflecting real-world clinical practice was to explore persistence of etanercept and other TNFi monotherapy after achieving remission/LDA while on combination therapy with csDMARDs. The study was based on data from the Corrona registry, representing real-world clinical practice in the United States for patients with RA.

## Materials and methods

### Study setting

Data for this analysis were obtained from the Corrona registry, an independent, prospective, observational cohort of patients with physician-confirmed diagnosis of RA, regardless of fulfillment of any classification criteria [19], currently recruiting patients from 177 private practices and academic sites with 752 participating rheumatologists across 42 US states. As of August 31, 2017 (the study cutoff), data for 46,542 adult patients with RA had been collected.

All participating investigators obtained full institutional review board (IRB) approval for human subject research. Sponsor approval and continuing review were obtained through a central IRB (New England Independent Review Board, NEIRB No. 120160610). For academic investigative sites without a waiver to use the central IRB, full board approval was obtained from the respective governing IRBs; documentation of approval was submitted to the Sponsor before initiating any study procedures. All registry participants provided written informed consent before participating.

### Study design and patient identification

This is a retrospective analysis of data from patients with RA enrolled in the Corrona registry. The study period was October 1, 2001, through August 31, 2017. Data from adults ≥ 18 years old with a physician-confirmed diagnosis of RA who had achieved remission or LDA on combination therapy with TNFi and csDMARD and then discontinued the csDMARD were analyzed. The index date was the date the patient discontinued csDMARD therapy and continued on TNFi monotherapy. To be included in the analysis, patients had to have a 6-month follow-up visit after the index date.

Remission was defined as Clinical Disease Activity Index (CDAI) score ≤ 2.8; LDA was defined as CDAI score > 2.8 and ≤ 10.0 [6, 20]. TNFis included adalimumab, certolizumab pegol, etanercept, golimumab, and infliximab; csDMARDs included methotrexate, hydroxychloroquine, leflunomide, and sulfasalazine.

Based on the treatment prescribed in the period immediately prior and up to the index date, patients were divided into two separate groups: those who were initially treated with etanercept and a csDMARD and those who were initially treated with other TNFis and csDMARD.

### Study outcomes

The primary outcome was the percentage of patients who remained persistent on monotherapy with the index TNFi. Persistence was defined as continuous use of the index monotherapy medication without any treatment gap ≥ 30 days during the 6- or 12-month period after the index date. Previous bDMARD experience has been shown to affect persistence [21–23]; therefore, persistence was explored separately for bDMARD-naïve and bDMARD-experienced patients. Demographic and clinical characteristics as predictors of persistence were assessed.

Secondarily, we evaluated the treatment patterns during the same 6- to 12-month period, including patients who discontinued their index TNFi, switched to another bDMARD, discontinued the TNFi and switched to csDMARD monotherapy, added csDMARD therapy, or escalated the TNFi dose. Finally, we considered the effect of prior exposure to bDMARD therapy and other factors on the odds of a patient continuing to persist on monotherapy by their 6-month visit.

### Statistical considerations

Baseline demographics and disease characteristics at the index date (i.e., the time of discontinuation of the csDMARD) were summarized separately for etanercept and other TNFis and were stratified by prior bDMARD exposure (naïve versus experienced).

Persistence was explored for the entire etanercept cohort and for the other TNFi cohort, and also separately stratified by prior bDMARD exposure. Chi-square tests were used to compare proportions, and *t* tests were used to compare continuous measures between patients on etanercept and those on other TNFi therapies. We also calculated a common value for the difference in persistence at the 6-month visit between etanercept and other TNFis across the bDMARD-naïve and bDMARD-experienced patients (stratified Mantel–Haenszel common risk difference). It was hypothesized that persistence according to prior bDMARD exposure for patients on monotherapy (etanercept versus other TNFi) was similar (Mantel–Haenszel tests calculated).

To identify the factors predictive of persistence, logistic regression models were fit; separate logistic regression models were fit for the etanercept and other TNFi monotherapy groups. Bivariate logistic regression models were first considered for selected variables, including demographic characteristics (age, sex, and race), clinical characteristics (duration of RA, anti-cyclic citrullinated peptide antibody [aCCP] status, and rheumatoid factor status), comorbidities (cardiovascular disease [i.e., coronary artery disease, myocardial infarction, coronary heart failure requiring hospitalization, acute coronary syndrome, unstable angina, cardiac revascularization procedure, cardiac arrest, and ventricular arrhythmia or other cardiovascular event], serious infection, or diabetes), and disease status (CDAI and physician global assessment [PGA]). Covariates that were statistically significantly associated with persistence on monotherapy (*p* < 0.05) were considered in multivariable models.

## Results

### Patients

**Fig. 1:**
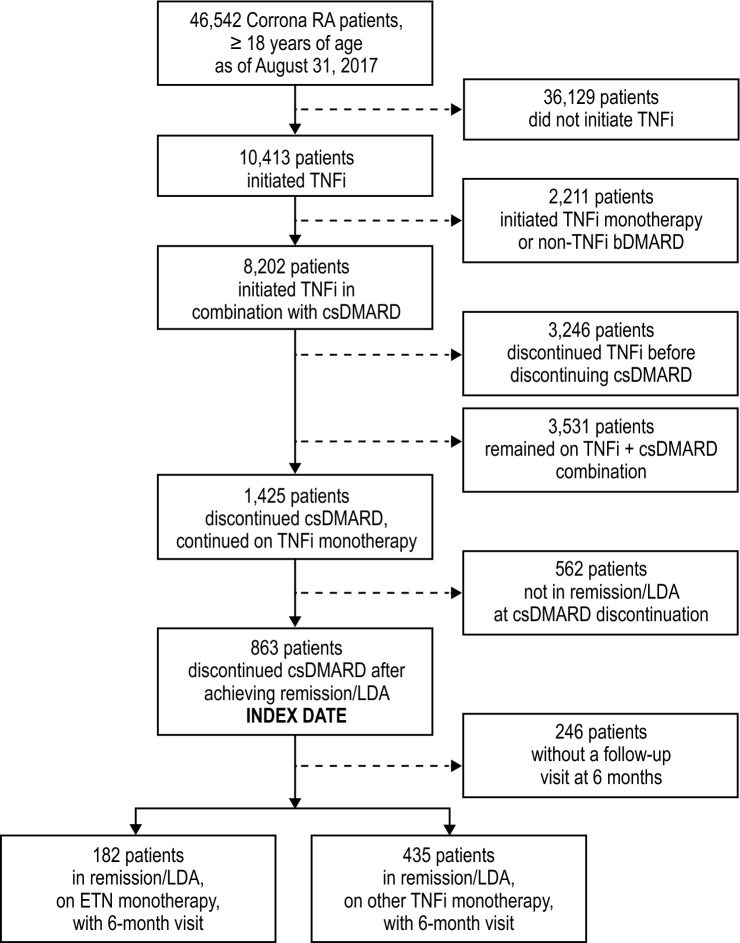
Patient identification. *bDMARD*, biologic disease-modifying antirheumatic drug; *csDMARD*, conventional synthetic disease-modifying antirheumatic drug; *ETN*, etanercept; *LDA*, low disease activity; *RA*, rheumatoid arthritis; *TNFi*, tumor necrosis factor inhibitor

Of the 46,542 adult patients with RA ever enrolled in the Corrona database, 10,413 patients were on a TNFi at the time of the analysis. 8202 patients had received a TNFi as part of a combination regimen with a csDMARD. Of these patients, 863 were in remission/LDA at the time the csDMARD was discontinued (index date); the 617 patients with a 6-month follow-up visit were included in this analysis. Of these, 182 patients were on etanercept monotherapy and 435 patients were on monotherapy with other TNFi therapies (Fig. 1).

Among patients on etanercept monotherapy and other TNFi monotherapy, 146 of 182 (80.2%) and 274 of 435 (63.0%) patients, respectively, had not received prior treatment with another bDMARD at any time during their enrollment in the Corrona database before initiation of the index biologic. When stratifying these patients, baseline characteristics were similar (Tables 1 and 2) except bDMARD-naïve patients on etanercept monotherapy were younger (mean, 55.5 versus 58.6 years; *p* = 0.024), had a longer time in remission/LDA before discontinuation of the csDMARD (mean, 19.0 versus 15.9 months; *p* = 0.202), and had a lower prevalence of cardiovascular disease (as determined at the index date: 0.7% versus 4.4%; *p* = 0.04) than bDMARD-naïve patients on other TNFi monotherapy, although the latter outcomes were based on few events. Biologic DMARD-experienced patients on etanercept (*n* = 36) and other TNFi therapy (*n* = 161) also had similar baseline characteristics (Tables 1,2), except fewer bDMARD-experienced patients on etanercept monotherapy had received > 1 prior bDMARDs (8.3% versus 27.3%; *p* = 0.016), patients on etanercept had been on bDMARD therapy longer than those on other TNFi therapy before the index visit (mean, 8.7 versus 5.7 months; *p* = 0.028) and had a shorter time in remission/LDA before the discontinuation of the csDMARD (mean 10.6 versus 18.4 months; *p* = 0.091), and a greater proportion of patients on etanercept monotherapy were currently using prednisone (25.0% versus 9.9%; *p* = 0.024).

**Table 1:** Baseline demographic and clinical characteristics among bDMARD-naïve and bDMARD-experienced patients at index date

	bDMARD-naïve patients		bDMARD-experienced patients	
	ETN (*n* = 146)	Other TNFi (*n* = 274)	ETN (*n* = 36)	Other TNFi (*n* = 161)
Age, mean (SD), years [*N*]	55.5 (13.0) [146]*	58.6 (13.7) [274]*	54.8 (12.4) [36]	57.6 (13.1) [161]
Female, *n* (%) [*N*]	106 (72.6) [146]	194 (71.1) [273]	30 (83.3) [36]	117 (72.7) [161]
White, *n* (%) [N]	127 (87.0) [146]	233 (85.0) [274]	29 (80.6) [36]	134 (83.2) [161]
Duration of RA, mean (SD), years [*N*]	8.8 (8.5) [143]	8.8 (8.2) [273]	11.5 (9.1) [36]	11.8 (9.2) [157]
RF positive, *n* (%) [*N*]	69 (72.6) [95]	152 (76.4) [199]	19 (82.6) [23]	62 (66.7) [93]
aCCP antibody positive, *n* (%) [*N*]	34 (55.7) [61]*	93 (72.1) [129]*	13 (86.7) [15]	42 (71.2) [59]
Comorbidities, *n* (%) [*N*]^†^				
Cardiovascular disease	1 (0.7) [146]*	12 (4.4) [274]*	1 (2.8) [36]	8 (5.0) [161]
Malignancy	9 (6.2) [146]	19 (6.9) [274]	4 (11.1) [36]	17 (10.6) [161]
Serious infection	4 (3.3) [121]	7 (3.1) [229]	1 (3.1) [32]	7 (5.1) [138]
Diabetes	5 (3.4) [146]	11 (4.0) [274]	3 (8.3) [36]	13 (8.1) [161]
mHAQ, mean (SD) score [*N*]	0.2 (0.4) [144]	0.2 (0.4) [266]	0.3 (0.4) [36]	0.3 (0.3) [157]
CDAI, mean (SD) score	3.7 (2.9) [146]	3.5 (3.0) [274]	5.0 (3.0) [36]	4.4 (2.9) [161]
CDAI category, *n* (%)				
Remission	68 (46.6)	147 (53.6)	11 (30.6)	59 (36.6)
LDA	78 (53.4)	127 (46.4)	25 (69.4)	102 (63.4)
PGA, mean (SD) score	7.7 (7.9)	7.0 (9.1)	10.5 (10.4)	8.9 (8.8)
Current prednisone use, *n* (%)	15 (10.3)	29 (10.6)	9 (25.0)*	16 (9.9)*
No. prior bDMARDs, *n* (%)				
0	146 (100)	274 (100)	0	0
1	0	0	33 (91.7)*	117 (72.7)*
> 1	0	0	3 (8.3)*	44 (27.3)*

*aCCP*, anti-cyclic citrullinated peptide; *bDMARD*, biologic disease-modifying antirheumatic drug; *CDAI*, Clinical Disease Activity Index; *ETN*, etanercept; *mHAQ*, modified Health Assessment Questionnaire; *PGA*, physician global assessment; *RA*, rheumatoid arthritis; *RF*, rheumatoid factor; *TNFi*, tumor necrosis factor inhibitor

*p < 0.05 for ETN monotherapy versus other TNFi monotherapy

^†^History of or current disease as assessed at index date, during the evaluation of index and baseline characteristics

**Table 2:** Time on therapy before achieving remission/LDA and before discontinuing csDMARD among bDMARD-naïve and bDMARD-experienced patients

Time, mean (SD), months	bDMARD-naïve patients		bDMARD-experienced patients	
	ETN (*n* = 146)	Other TNFi (*n* = 274)	ETN (*n* = 36)	Other TNFi (*n* = 161)
Time on TNFi therapy before achieving remission/LDA	4.8 (5.0)	6.0 (11.5)	8.7 (9.2)*	5.7 (6.8)*
Time on csDMARD before achieving remission/LDA	19.1 (21.4)	19.7 (22.6)	27.7 (25.3)	23.1 (24.8)
Time in remission/LDA before discontinuation of csDMARD	19.0 (25.5)	15.9 (23.1)	10.6 (13.3)	18.4 (26.6)
Time on csDMARD before discontinuation of csDMARD	36.3 (32.7)	38.6 (32.7)	41.8 (31.9)	39.1 (32.3)
Time on combination therapy before discontinuation of csDMARD	23.0 (25.1)	26.1 (26.9)	22.8 (18.9)	21.8 (24.7)

*bDMARD*, biologic disease-modifying antirheumatic drug; *csDMARD*, conventional synthetic disease-modifying antirheumatic drug; *ETN*, etanercept; *TNFi*, tumor necrosis factor inhibitor

*p < 0.05 for ETN monotherapy versus other TNFi monotherapy

Outcomes for two cohorts of patients were explored: those with a 6-month visit and those with a 12-month visit. Of the 617 patients included in the 6-month cohort, 120 patients on etanercept monotherapy and 307 patients on other TNFi therapy were also included in a 12-month cohort.

### Persistence

Overall persistence among all patients at 6 months was 56% for those on etanercept monotherapy and 45% for those on other TNFi monotherapy (crude risk difference 11.2% [95% CI 2.6%, 19.8%]; *p* = 0.02). At 12 months, persistence among all patients on etanercept monotherapy (46%) was higher than that for those on other TNFi monotherapy (33%) at 12 months (crude risk difference, 12.6% [95% CI 2.3%, 23.0%]; *p* = 0.03).

For the 6-month cohort, when stratified by prior bDMARD exposure, a higher proportion of bDMARD-naïve patients on etanercept were persistent with their index bDMARD (58%) than those who were also bDMARD-naïve on other TNFis (46%; risk difference was 11.9% [95% CI 2.0%, 21.9%]; *p* = 0.02). Among bDMARD-experienced patients, the proportion persistent on etanercept (50%) at 6 months was numerically higher but not statistically significantly different from those persistent on other TNFis (43%; risk difference was 6.5% [− 11.5%, 24.6%]; *p* = 0.48). Similar results were seen in the 12-month cohort (Table 3),

**Table 3:** Monotherapy persistence on index TNFi

Persistent on monotherapy after discontinuing csDMARD, *n* (%)	ETN monotherapy	Other TNFi monotherapy	Risk difference % (95% CI)	*p* value
6-month cohort				
bDMARD-naïve	84 (58)	125 (46)	11.9 (2.0, 21.9)	0.02
bDMARD-experienced	18 (50)	70 (43)	6.5 (− 11.5, 24.6)	0.48
All patients (stratified)^a^			10.8 (2.1, 19.6)	0.02
12-month cohort				
bDMARD-naïve	46 (47)	72 (36)	11.1 (− 0.8, 23.0)	0.07
bDMARD-experienced	9 (41)	30 (28)	12.6 (− 9.7, 34.9)	0.24
All patients (stratified)^b^			11.4 (0.9, 21.9)	0.03

*bDMARD*, biologic disease-modifying antirheumatic drug; *csDMARD*, conventional synthetic disease-modifying antirheumatic drug; *ETN*, etanercept; *OR*, odds ratio; *TNFi*, tumor necrosis factor inhibitor

^a^Mantel–Haenszel test of homogeneity reveals that the risk differences between the bDMARD-naïve and bDMARD-experienced patients were not significantly different (*p* = 0.61)

^b^Mantel–Haenszel test of homogeneity reveals that the risk differences between the bDMARD-naïve and bDMARD-experienced patients were not significantly different (*p* = 0.85)

In the 6-month cohort, the risk differences in the stratum-specific estimates between etanercept and other TNFis were similar between bDMARD-naïve and bDMARD-experienced patients based on the Mantel–Haenszel test of homogeneity (*p* = 0.61; Table 3). The difference in persistence at the 6-month visit between etanercept and other TNFis across the bDMARD-naïve and bDMARD-experienced patients had a stratified Mantel–Haenszel common risk difference of 10.8% (95% CI 2.1%, 19.6%; *p* = 0.02). Similar results were seen in the 12-month cohort (Table 3).

### Predictors of remaining on monotherapy

According to multivariable models conducted separately for etanercept and other TNFi monotherapy, being in remission at the index date (i.e., before discontinuing the csDMARD) was the strongest predictor of maintaining monotherapy with etanercept or with other TNFis (data not shown). For patients in remission at the index date, persistence on monotherapy was nearly twice that of patients not in remission (i.e., those with LDA). Conversely, every 10-year increase in age, every 1-unit increase in CDAI score, and every 1-unit (on a scale of 1‒100) increase in PGA score were associated with decreased likelihood of persistence on both etanercept monotherapy and other TNFi monotherapy. History of cardiovascular disease was associated with decreased likelihood of persistence on other TNFi monotherapy in the multivariate model. Results from the bivariate analysis were similar**.**

### Treatment patterns

The most common change to RA therapy during the 6 months after index date was the reintroduction of a csDMARD, which was more common among patients on other TNFi monotherapies (45%) than on patients on etanercept monotherapy (35%), regardless of prior bDMARD exposure (Table 4). Similar results were observed at 12 months following index date.

**Table 4:** Post-index treatment patterns

	bDMARD-naïve		bDMARD-experienced	
Treatment pattern, *n* (%)	ETN monotherapy	Other TNFi monotherapy	ETN monotherapy	Other TNFi monotherapy
6-month cohort, *N*	146	274	36	161
Discontinued index bDMARD	1 (1)	2 (1)	1 (3)	0
Switched to bDMARD	6 (4)	8 (3)	2 (6)	8 (5)
Switched to csDMARD	2 (1)	5 (2)	0	2 (1)
Added csDMARD	50 (34)	119 (43)	14 (39)	77 (48)
Stopped all RA therapy	3 (2)	15 (5)	1 (3)	4 (2)
12-month cohort, *N*	98	201	22	106
Discontinued index bDMARD	0	0	1 (5)	1 (1)
Switched to bDMARD	5 (5)	8 (4)	0	6 (6)
Switched to csDMARD	3 (3)	4 (2)	0	1 (1)
Added csDMARD	44 (45)	102 (51)	12 (55)	62 (58)
Stopped all RA therapy	0	15 (7)	0	6 (6)

*bDMARD*, biologic disease-modifying antirheumatic drug; *csDMARD*, conventional synthetic disease-modifying antirheumatic drug; *ETN*, etanercept; *RA*, rheumatoid arthritis; *TNFi*, tumor necrosis factor inhibitor

## Discussion

In this study, we investigated the persistence of etanercept monotherapy and that of other TNFi monotherapy after achieving remission/LDA on combination therapy with a csDMARD (typically methotrexate). For the overall patient group, etanercept monotherapy was found to be associated with greater persistence at 6 and 12 months (56% and 46%, respectively) than observed for other TNFi monotherapy (45% and 33%, respectively), with an intergroup difference favoring etanercept of 11.2% at 6 months and 12.6% at 12 months.

It is well known in clinical practice and in clinical trials that patients who are bDMARD-naïve will have a greater response to TNFi therapy than those with prior exposure to bDMARD [24]; therefore, persistence stratified by prior bDMARD exposure is important. This study found greater persistence among bDMARD-naïve patients for both etanercept monotherapy and other TNFis monotherapy groups. In analyses stratified by prior bDMARD experience, the intergroup difference favoring etanercept remained similar: 10.8% at 6 months and 11.4% at 12 months.

The extent of persistence observed in this analysis was similar to that previously reported [25–27]. In bDMARD-naïve patients (the majority receiving concomitant treatment with a csDMARD) with a mix of inflammatory rheumatic diseases (~ 50% with RA), 12-month persistence of > 50% was reported in an Australian real-world study [27]. In bDMARD-experienced patients with RA, persistence rates of approximately 40% have been reported [25, 26].

Using multivariate analysis, we found that remission was the major predictor of longer persistence; patients who achieved remission on combination therapy versus those who achieved LDA were almost twice as likely to have persistence on monotherapy. Others have reported a similar association between remission and persistency [28, 29]. Increasing age, higher CDAI scores, and higher PGA scores were associated with a reduced likelihood of persistence. Of these, only lower PGA scores were associated with an increased likelihood of persistent remission in other studies [28].

The differences between etanercept and other TNFi monotherapy in ongoing treatment persistence are of interest. Concerns have been raised over the therapeutic implications of the development of neutralizing antidrug antibodies to bDMARDs [15, 16], which may be the underlying rationale for recommending continuation with methotrexate monotherapy once good disease control has been achieved on combination therapy. The development of neutralizing antidrug antibodies to TNFis other than etanercept have been reported [15, 16, 30–34], and may be one factor contributing to the different persistence rates detected in our study. Similarly, the higher discontinuation rates reported for other TNFis than for etanercept in long-term registry studies [35, 36] is consistent with our findings. Furthermore, we found that patients on etanercept monotherapy were less likely than those on other TNFi monotherapy to have a csDMARD added to treatment over 6 or 12 months to maintain disease control.

Overall, the findings from this study may help inform future guidelines on how best to manage patients who respond well to combination therapy [6]. Currently, the ACR and EULAR guidelines are not specific about whether continuation on a single agent after achievement of LDA or remission is considered appropriate [5, 6]. APLAR and the latest French guidelines recommend tapering or discontinuing the targeted therapy rather than the csDMARD [7, 37]. However, randomized clinical trials show that the withdrawal of etanercept in patients who had achieved disease control with combination therapy was associated with a worsening of disease control [38, 39].

### Strengths and limitations

As with any study using registry data, our study has strengths and limitations. One strength of the study is the use of real-world data from one of the largest RA registries in the world, which has been accumulating data from nearly every US state, including rural, urban, academic, and private practices. Another strength of the Corrona registry is the systematic collection of disease activity measures. The registry has a range of internal validity and reliability checks to ensure the accuracy and completeness of the measures of disease activity and disease severity, treatment, adverse event, and quality-of-life data entered at each visit [19].

In common with all observational studies, a limitation of this analysis is that patients were not randomly assigned to treatments; therefore, there is a risk of selection bias and confounding. Notably, the etanercept group was slightly younger, less likely to have had cardiovascular disease, and had less exposure to prior bDMARDs. We thus stratified for bDMARD exposure. Among the bDMARD-experienced group, etanercept patients were more likely to be current users of prednisone than other TNFi patients. This imbalance in prednisone use based on a small sample of 36 bDMARD-experienced etanercept patients is in contrast to the several-fold larger sample of bDMARD- naïve patients where prednisone use was similar for etanercept (*n* = 146) and other TNFi (*n* = 274) patients (10.3% versus 10.6%; *p* > 0.999). We note that we did not adjust explicitly for the number of prior bDMARDs received; however, the results reported are meant to describe the differences between the two groups, including the differences in the baseline factors that may determine treatment decisions and may influence persistency with monotherapy.

The generalizability of results is also a potential area for concern. However, previous analysis of the Corrona database has demonstrated that the demographics and comorbidity profiles of patients included in the database were similar to patients with RA not included in the database [40].

Finally, in our analysis, we did not account for the degree of disease activity before the initiation of combination therapy csDMARD plus TNFi therapy, a factor which may prove to be a predictor of successful transition to TNFi monotherapy. However, there was no difference between treatment groups in disease activity at the time of discontinuing the csDMARD therapy in patients with remission/LDA.

## Conclusions

This study provides real-world evidence that a subset of patients who achieved remission or LDA when treated with a combination of csDMARD and TNFi were able to successfully transition to TNFi monotherapy (etanercept or other TNFi). Persistence with TNFi monotherapy was twice as likely in patients who achieved remission versus those with LDA. In this real-world setting, etanercept monotherapy was associated with greater persistence than was observed for other TNFi monotherapy, and a lower likelihood of requiring reintroduction of a csDMARD.
